# Influence and Optimization of Diverse Culture Systems on Chicken Embryonic Stem Cell Culture

**DOI:** 10.3390/genes15111400

**Published:** 2024-10-30

**Authors:** Wenjie Ren, Jun Wu, Xiaohang Lu, Dan Zheng, Guangzheng Liu, Gaoyuan Wu, Yixiu Peng, Kai Jin, Guohui Li, Wei Han, Xiang-Shun Cui, Guohong Chen, Bichun Li, Ying-Jie Niu

**Affiliations:** 1 Joint International Research Laboratory of Agriculture and Agri-Product Safety, Ministry of Education of China, Yangzhou University, Yangzhou 225009, China; 2 Key Laboratory of Animal Breeding Reproduction and Molecular Design for Jiangsu Province, College of Animal Science and Technology, Yangzhou University, Yangzhou 225009, China; 3 Poultry Institute, Chinese Academy of Agricultural Sciences, Yangzhou 225125, China; 4 Department of Animal Science, Chungbuk National University, Cheongju 28644, Republic of Korea

**Keywords:** chicken, ESCs, optimization, cytokines, derivation

## Abstract

Background: The importance of embryonic stem cells (ESCs) in chickens is undeniable, as they can be applied across various fields, including animal modeling, developmental biology, cell fate research, drug screening, toxicity testing, and gene function studies. However, a widely applicable culture system for chicken ESCs has yet to be developed. Objectives: This study aimed to investigate the effects of different culture systems on the derivation and maintenance of chicken ESCs, with a focus on optimizing the selected culture conditions. Methods: To achieve this, we tested the effectiveness of various species-specific ESC media in the derivation and culture of chicken PGCs, while incorporating different small molecule compounds to optimize the process. The pluripotency and differentiation potential of the resulting ESC-like cells were also evaluated. Results: The combination of PD0325901, SB431542, and LIF (R2i+LIF system) was found to be effective in generating chicken ESC-like clones. Further experiments showed that enhancing the R2i+LIF system with cytokines such as SCF and FGF2 significantly extended the culture period and increased the passage number of chicken ESC-like cells. These ESC-like cells were characterized through positive alkaline phosphatase staining and the expression of pluripotency markers POUV, NANOG, and SOX2. Additionally, differentiation assays confirmed their ability to form the three germ layers. Conclusions: The newly developed culture system provides suitable conditions for the short-term culture of chicken ESCs. However, further optimization is required to establish a system that can sustain long-term maintenance.

## 1. Introduction

Embryonic stem cells (ESCs) are a valuable biological tool with distinct characteristics, having the ability to differentiate into various cell types and continuously proliferate and renew themselves. These cells play important roles in various fields, including animal modeling, developmental biology, cell fate research, pharmacological screening, safety assessment, and genetic function analysis [1,2,3,4]. While stable ESC culture systems have been successfully established in a limited number of species, such as mice, humans, and cattle [5,6,7], the same cannot be said for chicken ESCs. Despite extensive efforts by researchers, achieving a stable and efficient culture system for chicken ESCs with a chemically defined medium has proven elusive, as these systems typically rely on serum supplementation [8,9,10]. The use of defined culture components is essential for studying the self-renewal and differentiation functions of ESCs. However, publicly available, well-defined culture media are relatively rare and only established in a few laboratories, making them underutilized. These obstacles present challenges in establishing reliable culture conditions suitable for chicken ESCs.

Early studies have demonstrated the significance of extracellular matrix (ECM) proteins and growth factors in maintaining ESC cell properties [11,12]. For instance, FGF2 has been found to be crucial for the self-renewal of hESCs [13] and has also been observed to promote chicken pluripotent stem cell culture [14]. Meanwhile, in the culture of mouse ESCs, the intervention of the MEK and TGFβ signaling pathways using small molecule inhibitors PD0325901 and SB431542 (referred to as R2i) effectively supports self-renewal and pluripotency [15,16]. In chickens, it has been shown that the addition of R2i and LIF can efficiently form ESC-like clones in the primary culture, but rapid differentiation occurs in subsequent passages [10]. Research on pluripotent stem cells has led to the development of various culture systems aimed at supporting their derivation and maintenance across different mammalian species. For example, studies have shown that adding low levels of activin (3 ng/mL), along with XAV939 and RAR inhibitors (RARi), can induce the formation of “formative” stem cells, an intermediate pluripotent state between naïve and primed [17]. Additionally, expanded potential stem cells (EPSCs) have been established in pigs and humans, demonstrating developmental potency across all embryonic and extra-embryonic cell lineages [18], broadening the scope of their potential applications. Further research has elucidated that pluripotent stem cells can exist in at least three states—naïve, formative, and primed—each defined by distinct signaling requirements and developmental capacities. This framework has provided a clearer understanding of pluripotency and how it is regulated across species. These findings offer valuable insights into ESC culture across different species, supporting further exploration of their potential applications. Nonetheless, the unique genetic traits of each species result in notable differences in ESC culture requirements, leaving it uncertain if these systems are compatible with chicken ESCs.

Our study aimed to identify the fundamental culture components for chicken ESCs and gradually optimize these conditions to establish the most suitable culture environment, advancing the research on ESC growth and characteristics. The study results revealed that KnockOut Serum Replacement (KSR) can effectively substitute FBS, and successful derivation of ESC-like cells was achieved by simultaneously adding R2i, LIF, SCF, and FGF2 (RLSF culture system), leading to short-term proliferation. Through our identification process, we found that the cells obtained under these conditions still expressed pluripotency marker genes and exhibited positive alkaline phosphatase (AKP) staining characteristics. The formation of embryoid-like bodies demonstrated the potential of these cells to differentiate into the three embryonic germ layers. These results indicate that the RLSF culture system we established can efficiently derive ESCs in vitro. These findings offer valuable insights for optimizing culture conditions and further exploring ESCs, with the potential to advance this field.

## 2. Materials and Methods

All chemicals were obtained from GIBCO BRL (Grand Island, NY, USA) unless stated otherwise. LIF, PD0325901, SB431542, FGF2, SCF, activin A, CHIR-99021, desloratadine, and minocycline hydrochloride were purchased from MedChemExpress.

### 2.1. Fertilized Eggs and Animal Care

All experimental procedures were approved by the Experimental Animal Ethics Committee of Yangzhou University. Fertilized eggs for the experiments were obtained from Jiangsu Institute of Poultry Sciences.

### 2.2. Cell Culture Media

The basic medium for avian ESCs was composed of KO-DMEM, 1× B-27 supplement, 1× N-2 supplement, 1× GlutaMax, 1× NEAA, 0.1 mM β-mercaptoethanol (Gibco, USA), 1× EmbryoMax Nucleosides (Sigma, GER, Taufkirchen, Germany), 1 mM sodium pyruvate, and 1× penicillin/streptomycin (Solarbio, Beijing, China). To test the effect of serum or substitutes on ESC cultures, 15% KSR, 15% FBS or 5% bovine serum albumin (BSA) was added, and an additional 0.2% chicken serum (Gibco, USA) or 10 µg/ml ovotransferrin was added to assess their impact on ESC cultures.

The RLSF medium contained the Avian ESC basic medium as the basal medium, and 1 μM PD0325901, 10 μM SB431542, 10 ng/mL LIF, 6 ng/mL SCF, and 4 ng/mL of human FGF2 were added to the medium. Additionally, the avian ESC basic medium was supplemented with 25 ng/mL of activin A and 4 ng/mL of human FGF2 to form an activin A+FGF2 medium. For the 2i+LIF medium, the avian ESC basic medium was supplemented with 0.2 μM CHIR-99021, 1 μM PD0325901, and 10 ng/mL LIF. The CLDM medium was composed of avian ESC basic medium supplemented with 0.2 μM CHIR-99021, 10 ng/mL LIF, 2 μM desloratadine, and 2 μM minocycline hydrochloride. Lastly, the PSDM medium was formed by adding 1 μM PD0325901, 10 μM SB431542, 2 μM desloratadine, and 2 μM minocycline hydrochloride to the avian ESC basic medium.

### 2.3. Preparation of Feeder Layers

Buffalo rat liver (BRL) and STO cells were obtained from ATCC and maintained in feeder medium (DMEM with 10% Fetal Bovine Serum and 1× penicillin/streptomycin). To prepare feeder layers, BRL and STO cells were treated with 10 μg/mL mitomycin C for 2 h and subsequently frozen in liquid nitrogen. Prior to the feeder layer use, thawed and inactivated BRL or STO cells were seeded onto culture dishes pre-coated with 0.1% gelatin the previous day.

### 2.4. Isolation and Culture of Blastodermal Cells (BCs)

Fertilized eggs from Rugao yellow chickens were freshly collected and disinfected with 70% ethanol. BCs from the area pellucida of EGK.X stage embryos were then isolated as per the standard procedure [19]. Briefly, the eggshells were carefully opened using forceps to reveal the embryo. A sterile filter paper ring was placed on the embryo, and the surrounding area was cut along the outer edge of the ring to separate the embryo from the yolk. After lifting the embryo, it was washed with PBS to remove any remaining yolk, and BCs in the area pellucida were isolated under a microscope with a needle. These cells were gently dissociated by pipetting and centrifuged at 1500× *g* rpm for 5 min to obtain a cell pellet. BCs from each embryo were then cultured on mitotically inactivated feeder cells. The medium was refreshed daily, and after 2–3 days, ESC-like colonies were detached using Accutase (40506ES60, Yeasen, Shanghai, China) for 5–7 min. The colonies were subsequently plated in a 12-well dish prepared with mitomycin C-treated BRL or STO feeder cells.

### 2.5. Alkaline Phosphatase (AKP) Staining

The method previously described was followed in order to determine the activity of AKP [20]. Briefly, chicken ESC-like cells were rinsed three times with PBS and fixed in 4% paraformaldehyde (PFA, Solarbio, China) for 5–10 min at room temperature. The fixed cells were then stained with an AKP staining working solution for 30 min at room temperature. The staining working solution contained 100 mM Tris-buffer (pH 8.2–8.4) with 200 mg/mL naphthol AS-MX phosphate (Sigma, GER) and 1 mg/mL Fast Red TR salt (Sigma, GER). After staining, the cells were washed again with PBS to stop the staining reaction. Finally, the stained cells were observed using a microscope.

### 2.6. Formation and In Vitro Differentiation of Embryoid Bodies (EBs)

After treating the ESC-like cells (third passage) with Accutase, the cells were transferred to a 15 mL centrifuge tube and centrifuged at 1000× *g* rpm for 5 min. The supernatant was discarded, and the remaining cells were seeded into a culture dish to allow for 1 h of adhesion. The ESC-like cells were then collected from the supernatant, effectively removing feeder cells. Following another centrifugation at 1000× *g* rpm for 5 min, the cells were resuspended in avian ESC basic medium. Finally, the cells were plated in ultra-low-attachment multiwell plates (CLS3473, Corning, NY, USA) and incubated in a 37 °C, 5% CO_2_ incubator for 3 to 5 days.

### 2.7. RNA Isolation and Reverse Transcription

A total of one million chicken ESC-like cells were harvested and placed in a centrifuge tube. One milliliter of Trizol reagent (Vazyme, Nanjing, China) was added, and the mixture was allowed to sit at room temperature for 5 min. Chloroform was then introduced, and the sample was vigorously shaken for 15 s before being left to stand for 2 to 3 min. The mixture was centrifuged at 10,000× *g* rpm for 15 min at 4 °C. Following centrifugation, an equal volume of isopropanol was added, thoroughly mixed, and allowed to rest for 10 min. The sample underwent another centrifugation at 10,000× *g* rpm for 10 min at 4 °C, and the supernatant was discarded. To wash the RNA pellet, 75% ethanol was added, and the sample was centrifuged again at 10,000× *g* rpm for 5 min at 4 °C. After discarding the supernatant, the pellet was dried, and 30 µL of DNase/RNase-free distilled water was added to dissolve the RNA. RNA concentration was measured using a NanoDrop 2000 spectrophotometer (Thermo Scientific™, Waltham, MA, USA).

Following treatment with DNase I, total RNA was reverse-transcribed using the HiScript^®^ III RT SuperMix (+gDNA wiper) kit (Vazyme, China), adhering to the manufacturer’s guidelines. The reaction mixture included 1 µg of RNA, 4 µL of 4× gDNA wiper mix, and RNase-free distilled water was added to achieve a total volume of 16 µL. The mixture was gently pipetted and incubated at 42 °C for 2 min. Next, 4 µL of 5× HiScript III qRT SuperMix was added, and the reverse-transcription program was executed at 37 °C for 15 min, followed by 85 °C for 5 s to synthesize the cDNA.

### 2.8. PCR and Agarose Gel Electrophoresis

PCR was performed starting with an initial 5 min denaturation at 98 °C, followed by 35 cycles of amplification at 98 °C for 10 s, 60 °C for 15 s, and 72 °C for 30 s. A final extension was carried out at 72 °C for 5 min. The reaction used PrimeSTAR^®^ Max DNA Polymerase (Takara, Dalian, China), and the primer sequences are listed in Table 1. PCR products were resolved on a 1.5% agarose gel, stained with RedSafe Nucleic Acid Staining Solution (INtRON Biotechnology, Jungwon-gu, Republic of Korea), and visualized under UV light.

### 2.9. Quantitative Real-Time PCR (qRT-PCR)

qRT-PCR was conducted using the ChamQ Universal SYBR qPCR Master Mix kit (Vazyme, China) as per the manufacturer’s protocol. Each 20 µL reaction contained 10 µL of 2× ChamQ Universal SYBR qPCR Master Mix, 2 µL of cDNA, 0.4 µL each of forward and reverse primers, with DNase/RNase-free distilled water added to complete the volume. The amplification program included an initial 30 s step at 95 °C, followed by 40 cycles of 95 °C for 10 s, 60 °C for 30 s, and 72 °C for 15 s, concluding with a 5 min final extension at 72 °C. Target genes included *POUV*, *SOX2*, *NANOG*, *PAX6*, *HNF1A*, and *PPARA*, with *GAPDH* serving as the reference gene. Primers for each gene are provided in Table 1. The 2^−ΔΔCT^ method [23] was applied to analyze mRNA quantification data.

### 2.10. Immunofluorescence and Confocal Microscopy

ESC-like clones were washed three times with PBS, fixed with 4% paraformaldehyde (Solarbio, China) for 30 min at room temperature, and washed twice with PBS. Cells were then permeabilized using PBS with 0.5% Triton X-100 (Solarbio, China) for 15 min at room temperature and blocked in PBS containing 1.0% BSA for 1 h. The primary antibodies—anti-SSEA-1 (1:100; MC-480, DSHB, IA, USA), anti-EMA-1 (1:100; MC-480, DSHB, IA, USA), anti-C-KIT (1:100; 8380-01, SouthernBiotech, Birmingham, AL, USA), and anti-SOX2 (1:100; sc-398254, Santa Cruz, TX, USA)—were diluted in blocking solution and applied for overnight incubation at 4 °C. After three PBS washes, cells were treated with donkey anti-rabbit IgG or goat anti-mouse IgG (HuaBio, Hangzhou, China) for 1 h at room temperature, followed by a 10 min DAPI stain (Solarbio, China) and three additional PBS washes. The slide was then mounted with 10 µL anti-fluorescence quenching agent (Solarbio, China), sealed with cedar oleoresin, and visualized using a Zeiss LSM 710 Meta confocal microscope. Image processing was performed with the Olympus Fluoview 4.1a viewer (Olympus, Tokyo, Japan).

### 2.11. Statistical Analysis

Each experiment was conducted at least three times, with representative images displayed in the figures. Statistical analysis was carried out using the Student’s *t*-test or one-way ANOVA. Results were reported as mean ± SEM. Statistical significance was set at *p* < 0.05, with all calculations performed in SPSS software v.19 (SPSS, Inc., Chicago, IL, USA).

## 3. Results

### 3.1. Influence of Diverse Culture Systems on Chicken ESC Culture

**Figure 1:**
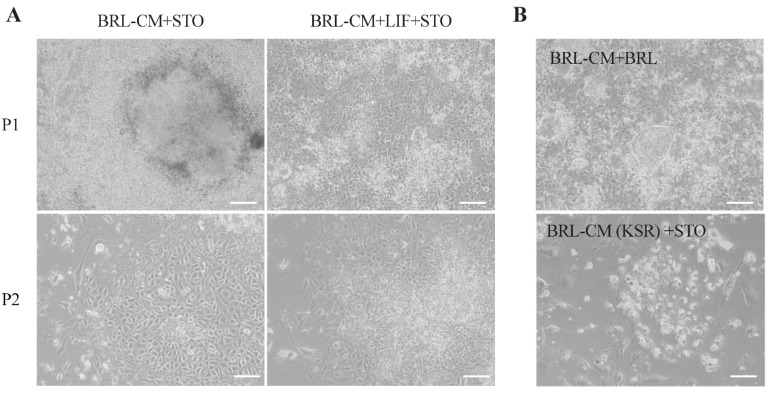
The impact of the BRL-conditioned medium-based ESC culture system on chicken ESC cultures. (**A**) Images showing clone formation by BCs under BRL-CM+STO with or without LIF addition. P1 and P2 indicate the first and second passages of the cells, respectively. (**B**) Derivation of ESC-like clones using BRL as feeder layer cells or KSR as a substitute for FBS in the BRL-CM+STO culture system. Scale bar represents 100 µm.

BCs were isolated from the EGK.X stage of fertilized eggs from Rugao Yellow Chickens. Initially, we cultured the BCs in the BRL condition medium (BRL-CM) and STO feeder layer conditions (BRL-CM+STO) [24]. After 3–5 days of culture, we obtained typical ESC-like cells; however, there were also a large number of differentiated cells present simultaneously. Additionally, ESC-like cells quickly differentiated after the first passages, and even the addition of LIF could not sustain their pluripotency (Figure 1A). Subsequently, we attempted to replace the feeder layer cells with BRL cells in the BRL-CM+STO culture system (Figure 1B), or to culture the BCs in the BRL-CM+STO system with FBS replaced by KSR (Figure 1B). Unfortunately, these conditions did not successfully derive ESC-like cells. This suggests that there are limiting conditions or missing crucial factors in these culture systems that hinder the maintenance of their pluripotency.

**Figure 2:**
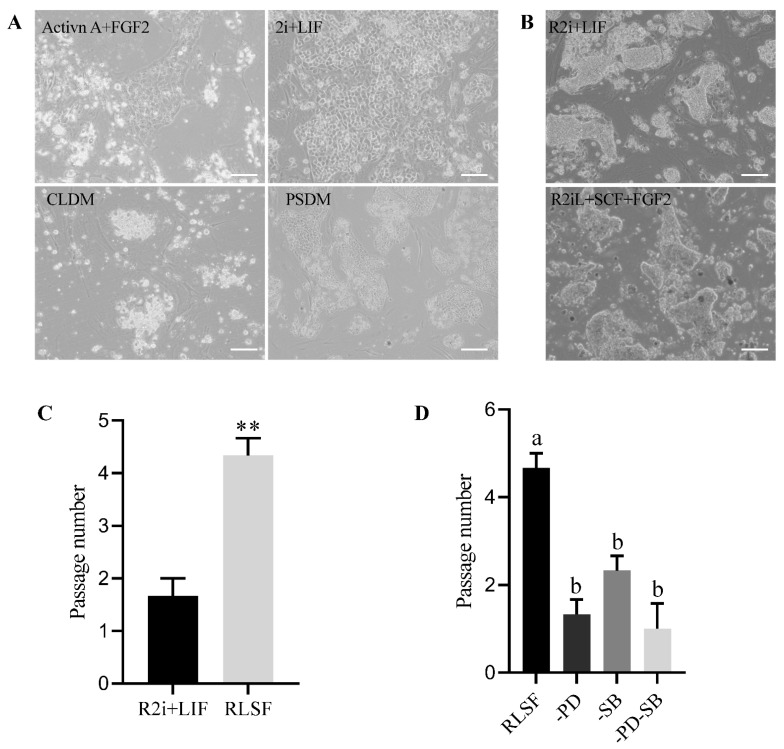
The influence of different pluripotent stem cell culture systems used in mammals on chicken ESC cultures. Scale bar represents 100 µm. (**A**) Representative images of clone formation by BCs under activin A+FGF2, 2i+LIF, CLDM, and PSDM culture systems. (**B**) Derivation and passage of chicken ESC-like cells in the R2i+LIF culture system. Scale bar represents 100 µm. (**C**) Adding SCF and bFGF to the R2i+LIF system significantly enhanced the passage numbers of chicken ESCs. ** indicates *p* < 0.01. (**D**) In the RLSF culture system, the absence of PD325901, SB431542, or both significantly reduces the passage ability of ESC-like cells. Different lowercase letters indicate significant differences, *p* < 0.05.

We further explored if the pluripotent stem cell culture systems established in mammals could be used for the derivation of chicken ESCs. After supplementing the base culture medium with activin A+FGF2, 2i+LIF, CLDM, PSDM, and R2i+LIF, respectively, we assessed the formation and proliferation of ESC-like cells. Our research found that only under the R2i+LIF condition could ESC-like cells be formed whereas other culture systems did not support the formation of ESC-like cells (Figure 2A,B). This study confirmed the significant role of R2i+LIF in supporting the derivation of chicken ESC-like cells.

### 3.2. Prolonging the Maintenance of Chicken ESCs by Adding SCF and bFGF

When we assessed the passage ability of ESC-like cells under the R2i+LIF culture system, the cells started to differentiate rapidly after the first or second passage (Figure 2B,C). Previous studies have demonstrated successful culture of chicken PGCs, EGCs, and ESCs through the addition of SCF, bFGF, and LIF [25,26]. The study also found that LIF cooperated with FGF2 to maintain the proliferation of ESCs [14]. Based on these results, we explored whether the addition of SCF and bFGF factors could prolong the maintenance of chicken ESC-like cells under the R2i+LIF condition. The results indicated that under the R2i+LIF+SCF+bFGF condition (RLSF system), chicken ESC-like cells remained undifferentiated for up to the fourth passage, significantly surpassing the R2i+LIF condition (Figure 2B,C). We further assessed the influence of the R2i compounds on ESC culture. Using the RLSF group as a control, we observed a significant decrease in passage ability when PD or SB was absent, or when both PD and SB were simultaneously lacking (Figure 2D). These findings suggest that R2i plays a critical role in maintaining the characteristics of ESC-like cells.

### 3.3. Maintaining the Pluripotency of Chicken BCs Under the RLSF Culture Conditions

**Figure 3:**
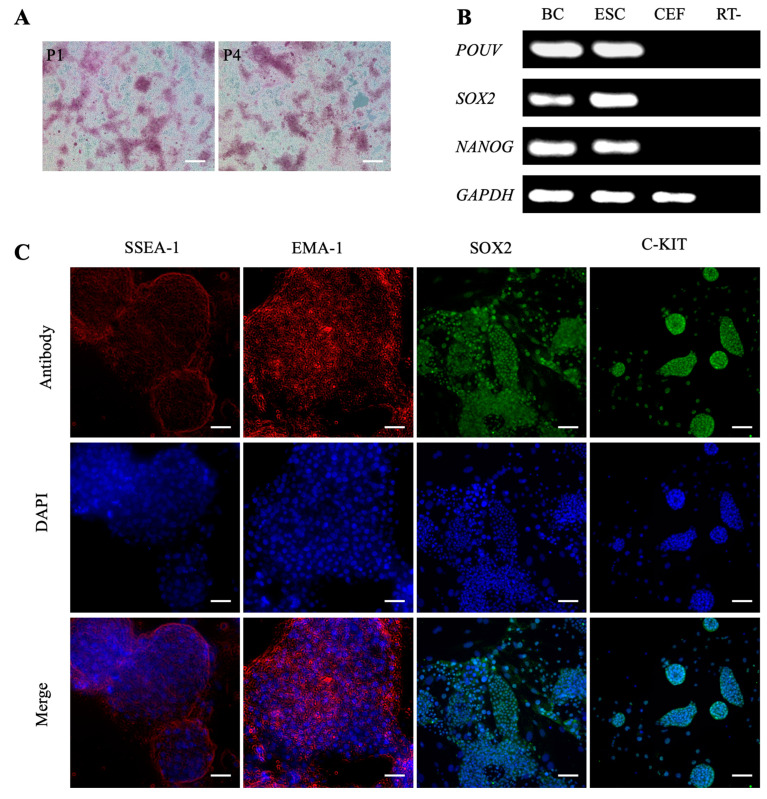
Examination of pluripotency in chicken ESC-like cells derived from the RLSF system. (**A**) Positive AKP staining in ESC-like cells derived from the RLSF system. P1 and P4 indicate the first and fourth passages of the cells, respectively. Scale bar represents 100 µm. (**B**) Expression of pluripotency genes in ESC-like cells. (**C**) Immunofluorescence staining analyses showing expression of SSEA-1, EMA-1, SOX2, and C-KIT proteins in ESC-like cells. Scale bar represents 40 µm.

**Figure 4:**
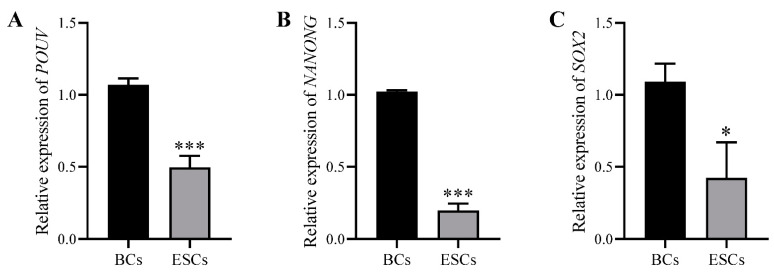
Comparison and analysis of the expression of pluripotency genes in BCs and ESC-like cells derived from the RLSF system. qRT-PCR analysis of *POUV* (**A**), *NANOG* (**B**), and *SOX2* (**C**) gene expression levels in ESC-like cells compared with BCs. * indicates *p* < 0.05. *** indicates *p* < 0.001.

To assess the effectiveness of the RLSF culture system in maintaining the biological characteristics of ESCs, we examined the AKP staining of the chicken ESC-like cells and observed positive results (Figure 3A). Following this, we conducted PCR and agarose gel electrophoresis experiments to analyze the expression of pluripotency genes in the cultured chicken ESC-like cells. The results revealed higher levels of *POUV*, *SOX2*, and *NANOG* genes compared to somatic cells (Figure 3B). To validate our findings, we performed immunofluorescence experiments, which indicated that the cultured ESC-like cells expressed the proteins SSEA-1, EMA-1, SOX2, and C-KIT, typical markers of the multipotent stem cells (Figure 3C). This further supported the notion that these cells still retained a certain degree of stem cell characteristics. However, qRT-PCR detection revealed that the expression levels of pluripotency genes *POUV*, *SOX2*, and *NANOG* in the cultured chicken ESC-like cells were significantly lower than in the BCs (Figure 4A–C). This observation may explain why the ESC-like cells could not be maintained for long-term passages.

### 3.4. ESC-Like Cells Cultured Under RLSF Conditions Exhibit Differentiation Potential

**Figure 5:**
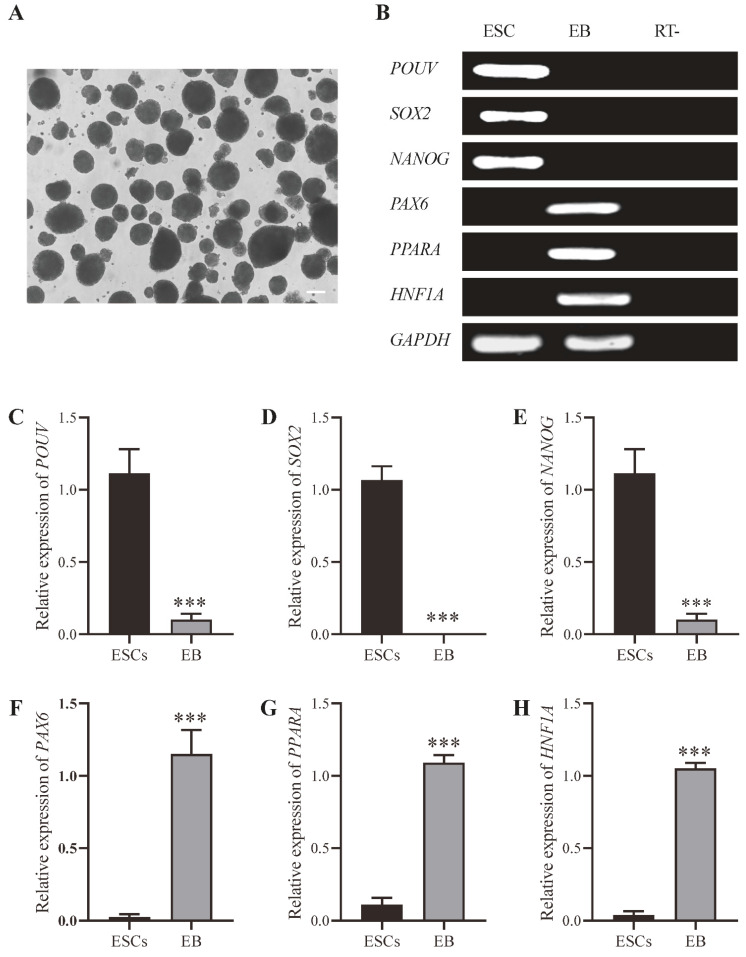
Differentiation potential of ESC-like cells cultured under RLSF conditions. (**A**) Images of embryoid bodies formed by ESC-like cells. (**B**) Upregulation of three germ layer genes and downregulation of pluripotency genes during EB formation. (**C**–**H**) qRT-PCR analysis of gene expression levels in ESCs compared with EBs. Scale bar represents 100 µm. *** indicates *p* < 0.001.

To assess the differentiation potential of ESC-like cells cultured under RLSF conditions, we performed EB experiments. We cultured the digested chicken ESC-like cells in the culture medium without PD0325901, SB431542, LIF, SCF, and FGF2 factors to induce their formation into EBs. We observed that EBs formed 5 days later (Figure 5A). Through PCR and agarose gel electrophoresis experiments, we observed that pluripotency genes were expressed in ESC-like cells, but their expression diminished after the formation of EBs (Figure 5B). Conversely, the three germ layer genes, *PAX6*, *PPARA*, and *HNF1A*, were exclusively expressed in EBs and not in ESC-like cells (Figure 5B). To further analyze these results, we conducted qRT-PCR. The results showed a significant increase in the expression of differentiation-related genes *PAX6*, *PPARA*, and *HNF1A* during the formation of EBs, while the expression levels of pluripotency genes *POUV*, *SOX2*, and *NANOG* decreased significantly (Figure 5C–H).

## 4. Discussion

Chickens not only play a crucial role in agriculture but have also become increasingly significant in the realms of cell fate research, gene function studies, and the development of vaccines and viral vectors in recent years [2,27,28]. Although primary cell cultures can replace fresh chicken eggs in some cases, achieving immortalization through genetic modification may be necessary, but their potential capacity may be lower compared to stem cells. The establishment of chicken ESC lines, on the other hand, presents multiple advantages, as ESCs have the ability to self-renew without the need for genetic modification, making them the best platform for establishing continuous cell lines [29]. However, different laboratories have used various methods in establishing continuous chicken ESC lines, and to date, there is still no widely used culture system [9,30,31]. This highlights the need for further research and standardization in this field.

Our main objective in this study was to establish a continuous culture system for chicken ESCs, which would serve as a powerful tool in cell fate research and gene function editing, laying the foundation for future large-scale applications. However, repeated attempts have shown that conventional culture conditions such as BRL-CM+STO and BRL-CM+BRL are not very efficient in the derivation of chicken ESCs, especially in the rapid differentiation after the first passage. When comparing with mature culture systems already applied in mammals, it was found that R2i+LIF played a crucial role in supporting chicken ESC-like cell proliferation. This is consistent with previous findings [10], providing a solid foundation for our subsequent research.

Previous studies have found that FGF2 can stimulate MEF to secrete TGFβ1, activin A, and gremlin [32]. In fact, TGFβ1 and activin A have been shown to work together with FGF2 to support the self-renewal of hESCs [33,34]. In addition, SCF plays an important role in the germ cells of chickens [35]. Based on this information, we explored whether combining these growth factors would have additional beneficial effects on maintaining the undifferentiated state of chicken ESCs. In our research, it was found that the combination of R2i+LIF+SCF+FGF2 had a positive effect on the maintenance of ESCs.

Subsequently, we identified the cell characteristics of the ESCs formed in the RLSF system to reveal the morphological characteristics, pluripotency, and differentiation potential of the derived ESCs [24,36]. Our results showed that ESCs derived in the RLSF culture system exhibited AKP positive staining. In addition, they expressed the pluripotency genes *POUV*, *SOX2*, and *NANOG*, consistent with previous research results [10]. Further immunofluorescence staining demonstrated the presence of specific proteins, further confirming the pluripotency of these cells by expressing C-KIT, EMA-1, SOX2, and SSEA-1 proteins. To verify if the cultured ESCs had the potential for multi-lineage differentiation, we performed EB experiments, confirming the potential of these cells to differentiate into three germ layer cells.

In conclusion, this study successfully established a short-term chicken ESC culture system, but there were still some differences and challenges compared to mammals. In the future, it is crucial to establish stable ESC culture conditions and delve into their molecular characteristics for the conservation and genetic resource research of chickens. These cells have tremendous potential in both agricultural and biomedical research. However, further research and optimization of culture systems remain a focus for future work to fully unleash the potential applications of these cells in various fields.

## Figures and Tables

**Table 1:** The primers used in the present study.

Gene	Primer Sequence	Annealing Temp.	Product Size (bp)	Reference
*POUV* F	GTTGTCCGGGTCTGGTTCT	60 °C	189	[21]
*POUV* R	GTGGAAAGGTGGCATGTAGAC			
*SOX2* F	GTGAACCAGAGGATGGACAGTTACG	60 °C	185	[22]
*SOX2* R	TGCGAGCTGGTCATGGAGTTG			
*NANOG* F	GGTTTCAGAACCAACGGATG	60 °C	121	[21]
*NANOG* R	GTGGGGGGTCATATCCAGGTA			
*PAX6* F	GAGAACCCACTATCCCGATGT	60 °C	200	—
*PAX6* R	GGTAAACGCTTGTGCTGAAAC			
*HNF1A* F	AGCCAGAACCTACTGAGCAC	60 °C	288	—
*HNF1A* R	GCTCCCCATGCTGTTTATCAC			
*PPARA* F	AATCACCCAGTGGAGCAGAAA	60 °C	266	—
*PPARA* R	CTCAGACCTTGGCATTCGTC			
*GAPDH* F	GAGGGTAGTGAAGGCTGCTG	60 °C	113	[21]
*GAPDH* R	CATCAAAGGTGGAGGAATGG			

## Data Availability

Data are contained within the article.
